# The Metabolic Flux Probe (MFP)—Secreted Protein as a Non-Disruptive Information Carrier for ^13^C-Based Metabolic Flux Analysis

**DOI:** 10.3390/ijms22179438

**Published:** 2021-08-30

**Authors:** Christian Dusny, Andreas Schmid

**Affiliations:** Department Solar Materials, Helmholtz Centre for Environmental Research (UFZ), Permoser Str. 15, D-04318 Leipzig, Germany; andreas.schmid@ufz.de

**Keywords:** ^13^C-labeling, metabolic flux analysis, metabolic flux probe, isotope mapping

## Abstract

Novel cultivation technologies demand the adaptation of existing analytical concepts. Metabolic flux analysis (MFA) requires stable-isotope labeling of biomass-bound protein as the primary information source. Obtaining the required protein in cultivation set-ups where biomass is inaccessible due to low cell densities and cell immobilization is difficult to date. We developed a non-disruptive analytical concept for ^13^C-based metabolic flux analysis based on secreted protein as an information carrier for isotope mapping in the protein-bound amino acids. This “metabolic flux probe” (MFP) concept was investigated in different cultivation set-ups with a recombinant, protein-secreting yeast strain. The obtained results grant insight into intracellular protein turnover dynamics. Experiments under metabolic but isotopically nonstationary conditions in continuous glucose-limited chemostats at high dilution rates demonstrated faster incorporation of isotope information from labeled glucose into the recombinant reporter protein than in biomass-bound protein. Our results suggest that the reporter protein was polymerized from intracellular amino acid pools with higher turnover rates than biomass-bound protein. The latter aspect might be vital for ^13^C-flux analyses under isotopically nonstationary conditions for analyzing fast metabolic dynamics.

## 1. Introduction

The quantification of intracellular metabolite flux distributions has become an integral tool for developing and optimizing industrially applied microbial whole-cell biocatalysts. Metabolic flux analysis based on ^13^C-stable isotope labeling is one key technology to decipher in vivo reaction ratios and rates of a metabolic network and its regulation at a genome-scale level in bacteria and yeast [1,2,3]. ^13^C-metabolic flux analysis delivers data on the actual utilization of the metabolic architecture, enabling the development of targeted metabolic flux redirection towards products in bioprocesses [4]. Flux analysis has been proven to help analyze cellular redox states, allowing to identify cofactor regeneration capacities in energy-demanding biotransformations [5,6].

The analytical principle of ^13^C-flux analysis relies on the specific labeling of the primary growth substrate with stable ^1^^3^C-isotopes [7]. Since amino acids are synthesized from the central carbon metabolism, the stable ^1^^3^C isotopes in the substrate are incorporated into the carbon backbone of proteinogenic amino acids at characteristic patterns, which depend on the employed precursor metabolites [8]. These characteristic isotope patterns can be analyzed via mass spectrometry or nuclear magnetic resonance and computationally translated into intracellular metabolic flux ratio distributions (METAFoR) based on whole-genome scale reaction network [9,10,11]. With extracellular rates, like specific growth rate, substrate uptake, and production rates, the flux ratios can be transformed into absolute intracellular rates [12]. Thus, ^13^C-metabolic flux ultimately enables one to quantitatively define metabolic phenotypes [13]. However, ^13^C-based metabolic flux analysis is designed to acquire isotope information from hydrolyzed biomass-bound protein [14]. Cell disruption denies any further analytical access to possible dynamic changes in intracellular metabolic flux distributions in set-ups where only low amounts of biomass are available or cells are immobilized, embedded, or encapsulated.

So far, only a few studies have demonstrated alternative concepts for ^13^C-based metabolic flux analysis. Sauer and coworkers developed a strategy to analyze metabolic flux distributions in mixed cultures [15]. An *Escherichia coli* wild-type strain and metabolic deletion mutants, synthesizing a recombinant protein, were co-cultured. The recombinant protein was separated from cell-bound protein with a selective purification protocol, which allowed discriminating metabolic flux distributions between wild-type and mutant cells in mixed cultivations. GC-MS analysis of both biomass-bound protein and recombinant reporter protein yielded consistent flux ratio distributions. Apart from this study, no alternatives sources to biomass-bound protein for obtaining ^13^C-labeling patterns from amino acids have been applied so far. However, cultivation concepts with biomass retention would benefit from analytical concepts that do not consume the available biomass for ^13^C-flux analysis. The ultimate increment of limited biomass availability can be found in single-cell analysis, becoming an increasingly important discipline within the systems biology framework [16].

Facing the steady development of mass spectrometry towards the analysis of femtomolar analyte amounts, microbial single cells come into reach as the following analytical targets for ^13^C-flux analysis [17,18]. Identifying single-cell metabolic phenotypes promises to open the new research field of single-cell fluxomics. In combination with the latest approaches in single cell-based genomics, transcriptomics, and proteomics, single-cell fluxomics would be a powerful tool to ultimately unravel microbial populations’ functional architecture and shed light on intrapopulation metabolic heterogeneity. The first steps towards microbial single-cell metabolomics have already been demonstrated by assessing the metabolic activity of individual *S. cerevisiae* based on ^13^C-isotope detection in adenosine triphosphate (ATP) with microarray-based matrix-assisted laser desorption/ionization mass spectrometry (MALDI-MS) [19]. However, single cells had to be quenched to preserve their metabolic state, and biomass was disrupted during laser desorption, forbidding further analysis of dynamics in single cells. Nevertheless, this study marks the beginning of a new era of microbial single-cell metabolomics. The demonstration of stable isotope detection inside single microbial cells might also move single-cell fluxomics into reach.

We aimed to establish a novel strategy for ^13^C-flux analysis, employing a secreted protein instead of biomass as a source for isotope labeling patterns in protein-bound amino acids. The rationale behind this investigation was to develop a method that enables flux analysis without consuming biomass in low cell density set-ups. This analytical principle, termed “flux probe concept”, was applied with different batch and continuous cultivation technologies, employing a recombinant, protein-secreting *Hansenula (Ogataea) polymorpha* yeast strain. Isotope labeling patterns in biomass-bound protein and secreted protein were quantitatively compared during metabolic steady-state to evaluate the flux probe concept. Dynamic isotope-labeling experiments in an isotopically nonstationary but metabolic steady-state were performed to reveal intracellular dynamics and protein turnover in biomass and secreted protein. Finally, the gathered isotope-labeling data were used to compute metabolic flux ratios and quantitatively translate this information into intracellular metabolic flux distributions.

## 2. Materials and Methods

### 2.1. Strains

Recombinant *Hansenula (Ogataea) polymorpha* RB11 conphys, expressing a consensus phytase gene (Mw = 61.5 kDa) under control of the host-intrinsic FMD promoter system with the expression vector pFPMT 121, was used throughout this study. The strain contains approximately 80 copies of the expression cassette and secretes the export-targeted consensus phytase protein into the extracellular medium upon promoter depression/induction. *H. polymorpha* strains were kindly donated by Dr. Michael Piontek (ARTES Biotechnology GmbH, Langenfeld, Germany).

### 2.2. Cultivation Workflow

All employed yeast strains were kept at −80 °C for long-term storage. For cryo-preservation, 1000 mL of an exponentially growing yeast culture in YPD complex medium were supplemented with 250 µL sterile 50% (*w*/*v*) glycerol solution, shortly vortexed and immediately frozen at −80 °C. For colony plating, small amounts of frozen cell suspension from cryo-stocks were scratched with a sterile platinum loop, streaked onto YPD agar plates, and incubated at 30 °C for 48 h. After visible colonies emerged, a single colony was picked with a sterile platinum loop from the agar plate to inoculate a sterile 15 mL falcon tube containing 5 mL of YPD medium. This preculture was incubated at 30 °C and 280 rpm in a rotary shaker (Shaker KS-15, Edmund Bühler GmbH, Bodelshausen, Germany). After cultivation in a complex medium, the overnight preculture was used to inoculate 25 mL of sterile mineral medium (SYN8 or SYN8-MES, supplemented with 0.5–2% carbon source in a 250 mL shake flask) again incubated overnight at 30 °C and 280 rpm shaking frequency. With the final overnight precultures, main cultures for batch and continuous cultivations were inoculated to an OD_600_ = 0.1, and the main cultures were used for subsequent experiments.

### 2.3. Continuous Cultivations for ^13^C-Based Metabolic Flux Ratio Analysis (METAFoR) and Metabolic Flux Analysis (MFA)

Glucose-limited continuous cultivations of *H. polymorpha* RB11 conphys were carried out using buffered SYN8-MES mineral medium containing 0.5% (*w*/*v*) of stable isotope-labeled 1-^13^C glucose or U-^13^C-glucose by default. Dilutions rates ranging from 0.05 to 0.2 h^−1^ were applied. All continuous cultivations were performed in a customized mini-chemostat system, or a 300 mL RALF stirred tank reactor (Bioengineering, Wald, Switzerland). For steady-state experiments in continuous chemostat cultivations (both in metabolic and isotopic steady-state), 1 mL of cell suspension was taken from the respective bioreactor tubes with a sterile syringe and immediately stored on ice in 1.5 mL Eppendorf cups. For experiments in metabolic and isotopic equilibrium, sampling was done after five residence times to guarantee steady-state conditions. The cell suspensions were subsequently centrifuged at 13,000 rpm and 4 °C for 10 min with a Biofuge fresco centrifuge (Kendro laboratory products GmbH, Langenselbold, Germany). The protein-containing cultivation supernatants were withdrawn and processed for protein purification and HPLC analysis. Supernatants were stored at 4 °C for not more than 2 days before downstream purification for GC-MS analysis. Freezing of cultivation supernatants was avoided since precipitation significantly hampered further protein purification from cultivation supernatants. The cell pellets from the cell suspension samples were washed twice with ice-cold dH_2_O and stored at −20 °C until further processing and analysis.

For sampling of biomass and protein-containing cultivation supernatants, dynamic analysis in metabolic steady-state, but in the nonstationary isotopic state, the medium was switched from naturally labeled glucose to 99.8% (mol/mol) U-^13^C glucose or vice versa. Samples of 1 mL were taken at the outlet tubes of the systems and not directly from the bioreactor tubes to maintain a metabolic steady state of the culture. In order to minimize changes in the labeling patterns by metabolic activity during the lengthy sampling procedure, the outlet tubes and Eppendorf cups for sample collection were immersed into an ice bath. After a sufficient sample volume was obtained, the samples were processed as described above for continuous cultures in metabolic and isotopic steady state.

### 2.4. Batch Cultivation for ^13^C-Based Metabolic Flux Ratio Analysis (METAFoR) and Metabolic Flux Analysis (MFA)

Batch cultivations were carried out in baffled 250 mL shake flasks, filled with 10% (*v*/*v*) of the nominal volume of the flask. For ^13^C-tracer experiments, the carbon source was either a mixture of 20% (mol/mol) U-^13^C glucose and 80% naturally labeled glucose, or 99.8% (mol/mol) 1-^13^C glucose in SYN8 mineral medium. The total glucose concentration for batch cultivation tracer experiments was 20 g L^−1^. The main culture with ^13^C-labeled glucose as carbon source was inoculated with an overnight preculture in mineral medium to an OD_600_ of 0.1 and cultivated at 30 °C and 280 rpm in a rotary shaker (Shaker KS-15, Edmund Bühler GmbH, Bodelshausen, Germany). Samples were not taken until the proportion of unlabeled biomass from the precultures inoculum dropped below 10% (*w*/*w*) of total biomass. Samples of 1 mL cell suspension were repeatedly taken in the early to the mid-exponential growth phase of the culture, and cell pellets and cultivations supernatants were processed as described above for continuous cultivations.

### 2.5. HPLC Analysis of Cultivation Supernatants

The quantification of extracellular metabolites, glucose, glycerol, acetate, ethanol, and phosphate was carried out with a LaChrom Elite HPLC system (VWR International GmbH, Darmstadt, Germany, and Hitachi High-Technologies Corporation, Tokyo, Japan) equipped with a 308R-Gel.H 300 × 8 mm column with a 048R-Gel.H 40 × 8 mm precolumn (Trentec Analysentechnik, Rutesheim, Germany). For elution, 5 mM H_2_SO_4_ was used as the mobile phase at a column temperature of 40 °C.

### 2.6. Protein Purification

For protein purification, sterile filtered cultivation supernatants from continuous chemostats and batch cultivations were transferred to 1 mL dialysis tubes with a cut-off of 8–10 kDa (Float-A-Lyzer G2 MWCO 8000-10000, Repligen, Ravensburg Deutschland) in order to reduce the content of low-molecular medium components and accumulated metabolites. Before dialysis, the tubes were preconditioned with 10% (*v*/*v*) ethanol solution for 15 min and excessively flushed with dH_2_O. Subsequently, individual samples were dialyzed against 1.5 L of Seralpur dH_2_O under gentle continuous stirring at RT (room temperature). During a period of 60 h, the dialysis buffer was changed after 4, 24, and 32 h. The desalted dialysate was then transferred into 1.5 mL Eppendorf tubes and concentrated in a standard vacuum centrifuge (Thermo Fisher Scientific, Waltham, MA, USA) at RT or a Savant SPD 121P SpeedVac concentrator (Thermo Fisher Scientific, Waltham, MA, USA) equipped with an RC 6 vacuum pump (Vacuubrand GmbH & Co. KG, Wertheim, Germany) and an RVT4104 refrigerated vapor trap (Thermo Fisher Scientific, Waltham, MA, USA) for 24–36 h. Besides dialysis, protein from cultivation supernatants was also purified with ultrafiltration. For this purpose, 3 mL of cell-free cultivation supernatant was concentrated with Millipore Amicon^®^ Ultra 15–30 k centrifugal filter (Merck Millipore Corporation, Burlingtoin, MA, USA) with a size cut-off of 15–30 kDa. The supernatant was pushed by centrifugal force through the filter membrane, employing a Heraeus Multifuge 1 S-R with TTH 400 rotor (Thermo Fisher Scientific, Waltham, MA, USA). The centrifugation step lasted 15 min at 4700 rpm and 4 °C and yielded 200 µL of concentrated protein solution. The protein solution was dried with a Savant SPD 121P SpeedVac concentrator (Thermo Fisher Scientific, Waltham, MA, USA) equipped with an RC 6 vacuum pump (Vacuubrand GmbH & Co. KG, Wertheim, Germany) and an RVT4104 refrigerated vapor trap (Thermo Fisher Scientific, Waltham, MA, USA) for 24–36 h. After drying, the protein residues were processed for GC-MS analysis.

### 2.7. Protein Quantification According to Bradford

Protein concentrations in cultivation supernatants and dialysates were measured with Bradford protein assay solution (Quick Start Bradford 1 × Dye Reagent, Bio-Rad Laboratories GmbH, Munich, Germany) according to the colorimetric assay procedure developed by Bradford et al. [20]. Before every experimental series of Bradford assays, calibration curves were determined with purified BSA (Bovine serum albumin) dissolved in SNY8 or SYN8-MES mineral medium to account for changes in the reactivity of the Bradford solution towards dissolved protein. The assay was typically carried out with 50 μL or 200 μL protein solution/cultivation supernatant added to 950 μL or 800 μL Bradford dye reagent in disposable polymer cuvettes, respectively. The sample preparations were covered with snippets of paraffin film, 10× carefully inverted, incubated for 10 min at RT, inverted 10 times again, and incubated for two more minutes at RT. The optical density OD595 of the samples was immediately measured with a Genesys 20 Spectronic photometer (Thermo Fisher Scientific, Waltham, MA, USA). If necessary, cultivation supernatant was appropriately diluted to enable measurement within the linear range of the assay.

### 2.8. SDS-PAGE Analysis

For the identification of intracellular and extracellular proteins from biomass pellets and cultivation supernatants, SDS-PAGE (sodium dodecyl sulfate polyacrylamide gel electrophoresis) according to the protocol published by Laemmli et al. was used [21].

### 2.9. Protein and Biomass Processing for GC-MS Analysis

For protein hydrolysis and liberation of protein-bound amino acids, dried protein residues or biomass pellets (approximately 1 mg CDW) were resuspended in 150 µL 6 M hydrochloric acid (HCl) and incubated at 105 °C for 18 h. After this, excessive HCl was evaporated, and samples were dried at 85 °C. Before GC-MS (gas chromatography-mass spectrometry) analysis, the dried protein hydrolyzates were dissolved in 30 µL of acetonitrile. Then 30 µL of MBDSTFA (*N*-methyl–*N*-tert-butyldimethylsilyl trifluoroacetamide) was added for the derivatization of the amino acids. The derivatization reaction was performed for 90 min at 85 °C. The distribution of 1-^13^C isotopomers within the carbon backbone of the proteinogenic amino acids was determined by GC-MS analysis. 1 µL of derivatized biomass hydrolysate were injected into a CP-3800 gas chromatograph coupled to a 1200 Triple Quad MS/MS, equipped with a 30 m HP55ms column, ID 250 µm (Varian Inc., Palo Alto, CA, USA). As a carrier gas, helium was used at a volumetric flow rate of 1 mL min^−1^. Injector and ion source temperatures were fixed at 250 °C; the transfer line was heated to 280 °C. The ionization of the eluted compounds was carried out in EI ionization mode at −70 eV. The PTV (programmed temperature vaporization) injector was operated at three different split ratios (split-less, 1:5 (low split), and 1:20 (high split)) were used to enable optimal signal intensities according to the varying total amino acid concentration of the analyzed samples. An initial temperature of 150 °C was maintained for 2 min upon sample injection, then increased to 249 °C at a rate of 3 °C min^−1^, again increased to 280 °C at a rate of 20 °C min^−1^ and kept constant for 1.45 min. All MS analyses were carried out in SIM (selected ion monitoring) mode to enhance the sensitivity of the quadrupole detection by predefining analysis frames with mass/charge (*m*/*z*) ratios of the expected amino acid fragments and dwell times adjusted to minimize calibration shifts for low mass fragments.

### 2.10. Stable ^1^^3^C-Isotope-Based Metabolic Flux Ratio Analysis (METAFoR)

For the computational estimation of intracellular fluxes and flux ratios, the software module FiatFlux 1.61 [9] was used within a MatLab 2009b (Mathworks, Natick, MA, USA) software environment. A stoichiometric model of the central carbon mechanism in yeast was adapted to *H. polymorpha* for ^13^C-constrained metabolic flux analysis [2,22]. The final model consisted of 31 intracellular metabolites and 43 unknown fluxes. The precursor demand for DNA, RNA, Protein and Lipid synthesis was calculated based on the biomass composition of methylotrophic yeast according to Carnicer et al. and implemented into the NETTO module of FiatFlux [22]. FractionaL^−1^^3^C-labeling patterns in the carbon backbone of the amino acids from GC-MS measurements were corrected for naturally occurring stable isotopes and the percentage of unlabeled biomass (for batch cultivations) before using the data in FiatFlux [9,23]. METAFoR analysis was performed with the RATIO software module of FiatFlux under consideration of the stable isotope labeling patterns in the backbone of the employed carbon source (1-^13^C or U-^13^C glucose). The experimental error was implemented according to Fischer et al. [14,24]. Further information on the metabolic model and flux ratio calculations can be found in the Supplementary Information.

## 3. Results

**Figure 1:**
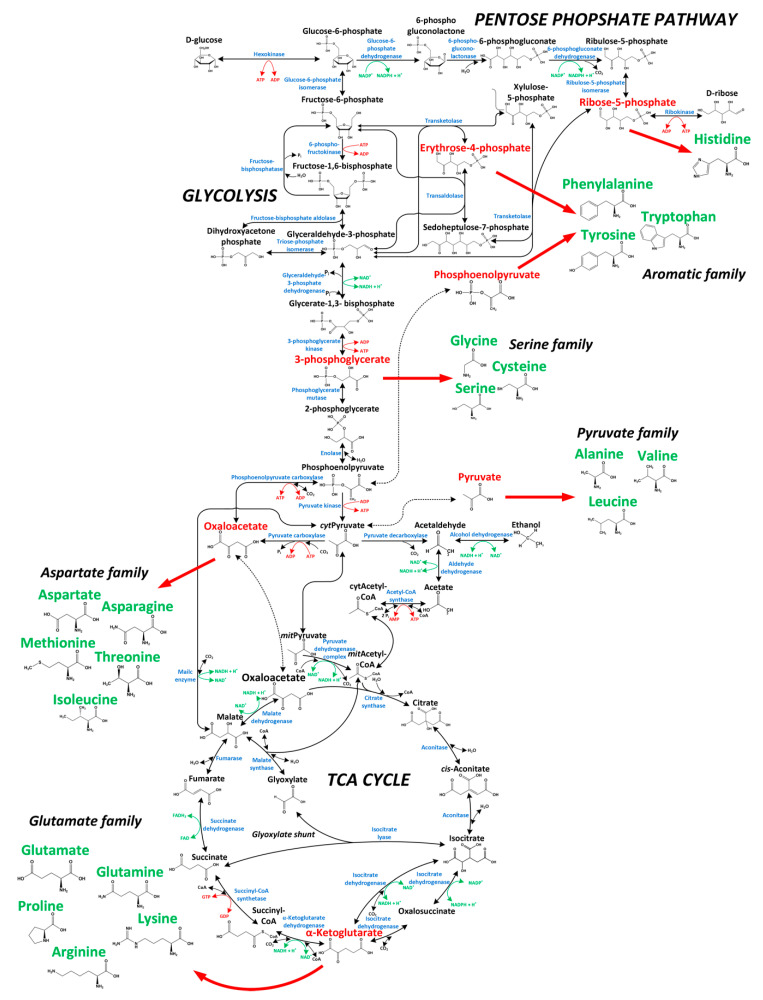
Central carbon metabolism of *H. polymorpha* RB11 conphys with precursor metabolites (red) for the biosynthesis of the 20 proteinogenic amino acids (green).

**Figure 2:**
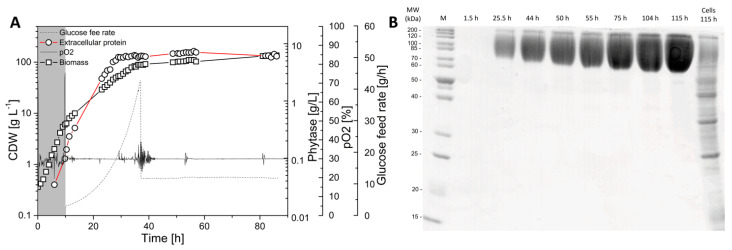
Production of consensus phytase with *H. polymorpha* RB11 conphys. (**A**) High cell density, glucose-limited fed-batch cultivation of *H. polymorpha* RB11 conphys with glucose as the sole carbon source in SYN-6 high cell density mineral medium. After an initial batch phase, an exponential glucose fed batch was applied to initiate the production phase. The specific growth rate µ of the cells was limited by the glucose feed to ensure continuous activity of the FMD promoter system. A final protein concentration of 7.4 g L^−1^ was reached. Glucose feed rates, pO_2_, biomass, and protein titers were followed for 85 h of cultivation time. (**B**) SDS-PAGE of untreated, extracellular cultivation supernatants from high cell density, glucose-limited fed-batch cultivation of *H. polymorpha* RB11 conphys. Based on a densitometry analysis, the protein purity of consensus phytase in the supernatant was determined as 97%. Glycosylation of the protein resulted in increased molecular weight of the consensus phytase, resulting in band smearing. The lanes correspond to the respective sampling time points in the course of cultivation. The lane to the very right was loaded with cell extract. (M = Marker protein ladder).

The primary goal of this study was to investigate whether secreted protein provides equivalent labeling information as biomass-bound protein for ^13^C-based metabolic flux analysis. Moreover, the resolution of intracellular dynamics in protein and amino acid turnover in overproduced protein and biomass was the focus of the study. Labeling kinetics in secreted protein and biomass were therefore analyzed in order to judge this. Here, we used a protein-secreting methylotrophic yeast, *H. polymorpha* RB11 conphys, as a model organism to verify the flux-probe concept. The central carbon metabolism and biosynthesis pathways for the 20 proteinogenic amino acids constituted the analytical target of this study and are illustrated in Figure 1. As can be seen from the pathway map, it is necessary to measure ^13^C labeling patterns in at least seven different amino acids synthesized from the seven different precursor metabolite pools to resolve the intracellular flux ratio distribution fully. The employed yeast strain *H. polymorpha* RB11 conphys synthesized and secreted the recombinant consensus phytase gene under the methanol-inducible FMD promoter system [25]. Secreted phytase served as the flux probe and was used as an additional source of amino acids besides biomass-bound protein. Notably, the FMD promoter is also highly active under glucose-derepression conditions at approximately 80% of methanol-induced activity [26]. The expression cassette was stably integrated into the genome and is present at a high copy number of approximately 80. This particular expression system is one of the most efficient platforms for producing heterologous protein, which was demonstrated to reach extracellular protein titers of up to 14 g L^−1^ with glucose as the sole carbon source [27]. We verified the protein production capacity of *H. polymorpha* RB11 conphys in high cell density glucose-limited fed-batch cultivation experiments. *H. polymorpha* RB11 conphys secreted recombinant consensus phytase at a high purity of 97% and a maximal specific production rate of 8.35 mg gcdw h^−1^ (see Figure 2). On account of its exceptionally high protein productivity and secretion capacity, *H. polymorpha* RB11 conphys constituted an ideal platform for investigating the flux probe concept and was used for all further ^13^C-flux experiments.

### 3.1. Development of a Sample Processing Workflow for Isotope Mapping with Secreted Protein

**Figure 3:**
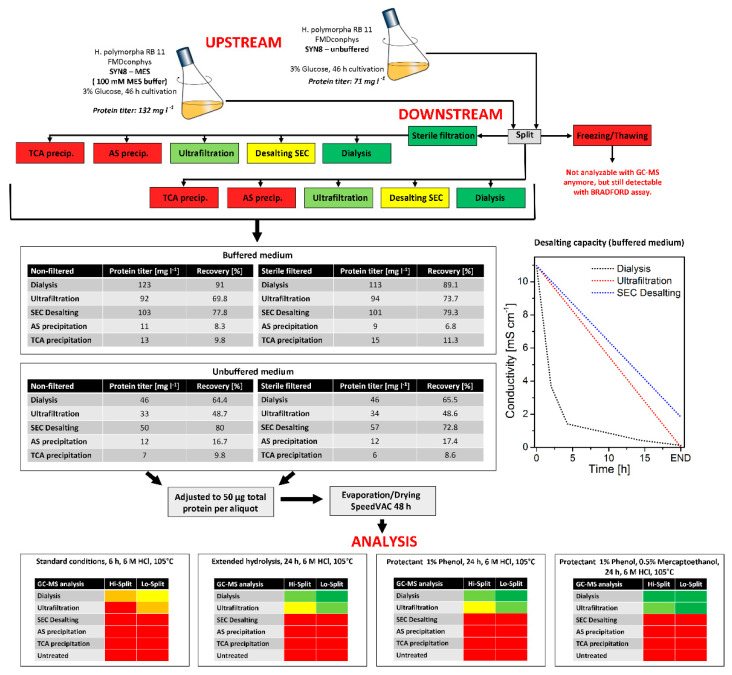
Evaluation of protein purification methods for secreted protein and subsequent ^13^C-isotope mapping via GC-MS.

A central prerequisite for applying the flux probe concept was proper processing of the secreted protein to access the ^13^C-labeling information stored in the respective carbon backbones of the amino acids via GC-MS analysis. Preliminary experiments revealed that the remaining medium compounds in the cultivation supernatants forbid a proper mass spectrometry analysis of the protein hydrolysate. Therefore, the secreted protein had to be purified before further processing. Five different protein purification methods, namely TCA (trichloroacetic acid) precipitation, AS (ammonium sulfate) precipitation, ultrafiltration, size-exclusion chromatography, and membrane dialysis, were compared with regards to protein recovery and desalting capacity (see Figure 3). During this evaluation, we found out that freezing and storage of the cultivation supernatants at −20 °C made protein analysis via GC-MS impossible, regardless of the employed purification method.

In contrast, the protein was still detectable in a Bradford assay and SDS-PAGE. Consequently, cell-free supernatants were immediately processed or stored at 4 °C for no longer than 24 h. Among the methods compared, dialysis turned out to be superior to the other investigated purification methods with a protein recovery of 65% (*w*/*w*) and desalting capacity of almost 100%. The conductivity of the cultivation supernatants was close to dH_2_O after four dialysis passages. In contrast, both precipitation methods had significantly lower protein yields of maximal 16.7% (*w*/*w*) and 9.8% (*w*/*w*). With size-exclusion chromatography, an excellent protein recovery of more than 80% (*w*/*w*) was possible, but the desalting capacity was insufficient for proper GC-MS analysis of the purified protein. The analysis procedure was further examined by hydrolysis efficiency. Four different protein hydrolysis methods with 6 M HCl were evaluated on the effect of hydrolysis time and the addition of protective agents for improved amino acid yields. With the addition of 1% (*w*/*v*) phenol and 0.5% (*w*/*v*) mercaptoethanol, preserving tryptophane from irreversible destruction and methionine and cysteine from oxidation, at a total hydrolysis time of 24 h at 105 °C, optimal results were obtained with protein from dialysis and ultrafiltration purification. All subsequent protein purification and processing in this study were therefore performed with dialyzed cultivation supernatants. The dried protein pellet (50 µg total protein) was hydrolyzed at 105 °C for 24 h in 6 M HCl, supplemented with 1% (*w*/*v*) phenol and 0.5% (*w*/*v*) mercaptoethanol. The concentration limit for a reproducible quantification of isotope patterns in the amino acid fragments was approximately 1 µg of hydrolyzed and derivatized protein per injection.

### 3.2. ^13^C-Isotope Mapping from Secreted Protein and Biomass in Aerobic Batch Cultivations (Metabolic and Isotopic Steady-State)

**Figure 4:**
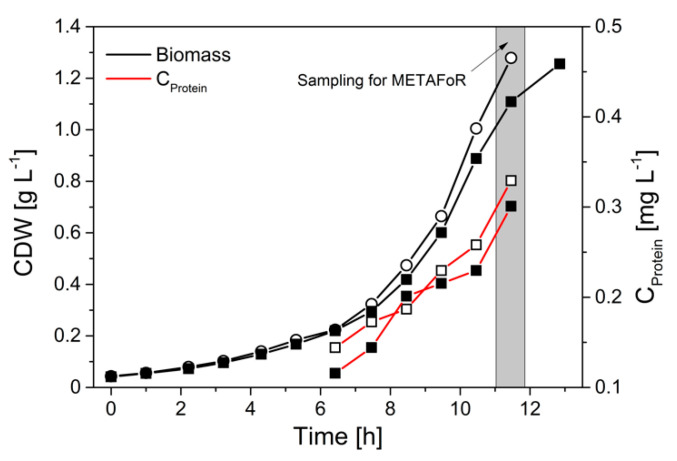
Growth of *H. polymorpha* RB11 conphys in aerobic batch culture with 5 g L^−1^ 100% (mol/mol) 1-^13^C glucose as sole carbon source. Biomass and extracellular protein from the cultivation supernatant were harvested for METAFoR in the late exponential growth phase.

With an optimized protein purification protocol at hand that allowed reproducible GC-MS analysis of the amino acids from secreted phytase protein, we next evaluated the flux probe concept in anaerobic batch cultivations of *H. polymorpha* RB11 conphys with 100% (mol/mol) 1-^13^C and 20% (mol/mol) U-^13^C glucose as sole carbon source, respectively (see Figure 4). The mixture of substrates with different isotopic labeling was employed to derive intracellular flux ratios. Biomass and cultivation supernatant were sampled 10.5 h after inoculation in the late exponential growth phase, obtaining secreted protein titers in the cultivation supernatants sufficient for GC-MS analysis. During the exponential growth phase, a metabolic and isotopic pseudo-steady state was assumed to prevail. The maximal protein productivity in batch cultivations was 50-fold reduced with a specific protein production rate of 237.1 µg gcdw h^−1^ compared to glucose-limited fed-batch cultivations due to glucose-repression of the FMD promoter.

**Figure 5:**
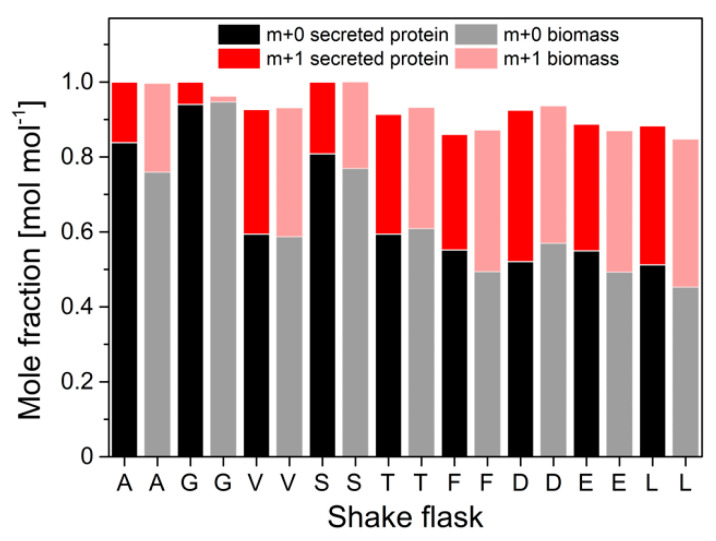
Molar fractions of m + 0 and m + 1 isotopomers in selected proteinogenic amino acids from hydrolyzed biomass and hydrolyzed secreted protein from *H. polymorpha* RB11 conphys. Cells were grown in aerobic batch culture with 5 g L^−1^ 100% (mol/mol) 1-^13^C glucose as the sole carbon source. Biomass and protein samples were taken in the mid-exponential growth phase after 11.5 h of total cultivation time. (A = alanine, G = glycine, V = valine, S = serine, T = threonine, F = phenylalanine, D = aspartate, E = glutamate, L = leucine).

Protein was purified from a 50 mL cultivation supernatant and hydrolyzed. The ^13^C-labeling patterns in the amino acid carbon backbone from the hydrolyzed secreted protein were derived from GC-MS analysis and compared to labeling patterns in amino acids from biomass-bound protein. The molar fractions of labeled (m + 1) and unlabeled (m + 0) m-57 amino acid fragments were calculated from GC-MS data and illustrated in Figure 5.

**Figure 6:**
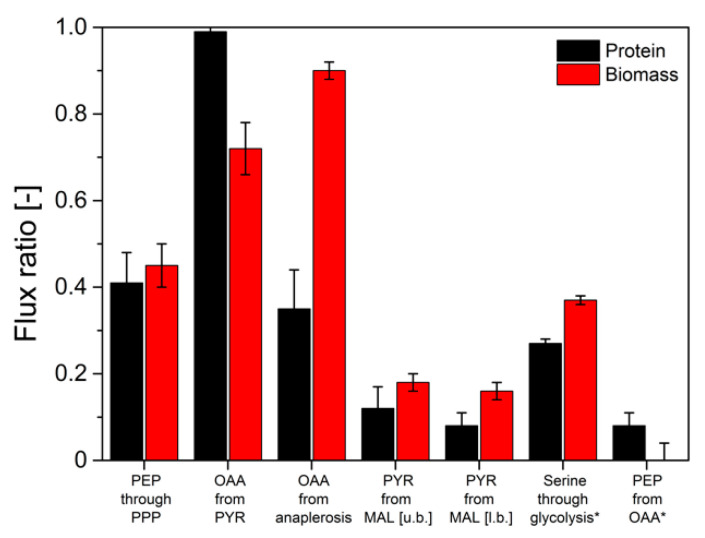
Comparison of intracellular flux ratios of *H. polymorpha* RB11 conphys in aerobic batch culture determined based on ^13^C-isotope mapping in amino acids from secreted protein and biomass with METAFoR analysis. The first five ratios were determined from labeling experiments with 20% (mol/mol) U-^13^C glucose, and 80% (mol/mol) naturally labeled glucose as a sole carbon source at a total concentration of 5 g L^−1^. Ratios marked with an asterisk were calculated from labeling experiments with 5 g L^−1^ 100% (mol/mol) 1-^13^C-glucose. (PEP = phosphoenolpyruvate, PPP = pentose phosphate pathway, OAA = oxaloacetate, PYR = pyruvate, MAL = malate, u.b. = upper bound, l.b. = lower bound).

Interestingly, significant differences in the abundance of labeled m + 0 and single ^13^C-labeled m + 1 fragments were measured between secreted protein and biomass-bound protein. Maximum differences of 14% (mol/mol) were determined for pyruvate-derived alanine, valine and leucine, and α-ketoglutarate-derived glutamate. Low deviations of 3% (mol/mol) were observed in the ^13^C-isotope of 3-phosphoglutarate-derived serine and glycine, as well as in phenylalanine and aspartate. In order to evaluate these differences and quantitatively assess their impact on the information about the actual intracellular pathway usage, a METAFoR analysis was performed. The flux ratios obtained from these analyses are displayed in Figure 6.

The determined flux ratios from secreted protein and biomass deviated by up to 61.1% for the flux of anaplerotic reactions to oxaloacetate. A deviation of 27% between secreted protein and biomass for the ratio from serine through glycolysis marked the highest complying flux ratio determined from batch experiments. Hence, comparably minor deviations in the determined labeling patterns resulted in significant deviations in the calculated intracellular flux ratio distributions. However, in previous experiments, the validity of flux information obtained from biomass-bound protein in pseudo-steady-state batch experiments has been proven [15]. Therefore, it could be concluded that flux ratios determined from isotope mapping in secreted protein do not reflect the actual intracellular flux distributions in *H. polymorpha* RB11 conphys. Thus, the flux probe concept is not applicable for ^13^C-based analysis of metabolic pathway activity in discontinuous cultivations.

### 3.3. ^13^C-Isotope Mapping from Secreted Protein and Biomass in Continuous Glucose-Limited Chemostat Cultivations (Metabolic and Isotopic Steady-State)

**Figure 7:**
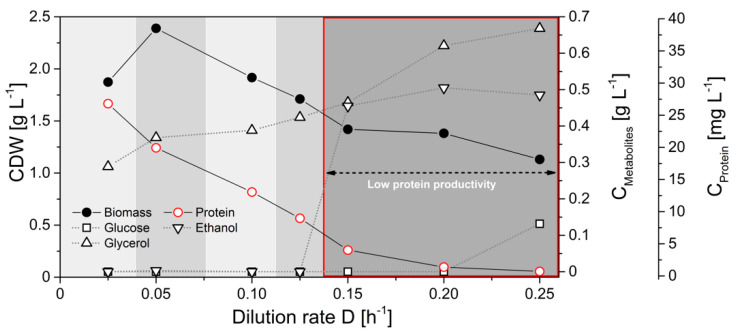
Cultivation profile of *H. polymorpha* RB11 conphys in aerobic continuous glucose-limited chemostats at different dilution rates with 0.5% (*w*/*v*) 100% (mol/mol) 1-^13^C glucose as the sole carbon source.

Next, we evaluated the flux probe concept in continuous glucose-limited chemostats. In contrast to batch cultivations, continuous chemostats enable maintaining a true metabolic and isotopic steady state at defined specific growth rates with continuous resupply of carbon source and nutrients and the removal of biomass and secreted metabolites. Physiological parameters of *H. polymorpha* RB11 conphys in glucose-limited continuous cultivations at different dilution rates ranging from D = 0.03 h^−1^ to d = 0.25 h^−1^ are illustrated in Figure 7.

**Figure 8:**
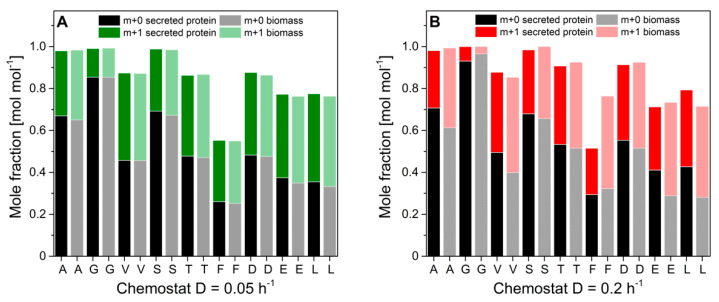
Molar fractions of m + 0 and m + 1 isotopomers in proteinogenic amino acids from hydrolyzed biomass and hydrolyzed secreted protein from *H. polymorpha* RB11 conphys cultivated at dilution rates (**A**) D = 0.05 h^−1^ and (**B**) D = 0.2 h^−1^. Cells were grown in aerobic continuous glucose-limited chemostats with 100% 1-^13^C glucose as the sole carbon source. (A = alanine, G = glycine, V = valine, S = serine, T = threonine, F = phenylalanine, D = aspartate, E = glutamate, L = leucine).

Notably, the specific protein production rate was governed by the applied dilution rate and increased with glucose limitation. Specific protein production rates ranged between 0.19 mg gcdw^−1^ h^−1^ and 1.03 mg gcdw^−1^ h^−1^. Interestingly, protein productivity was 8-fold decreased than maximal protein productivities during glucose-limited high cell density fed-batch cultivations. Biomass concentrations were rather constant and independent of the dilution rate. Ethanol production was detectable at dilution rates above 0.125 h^−1^, indicating anaerobic induction from fully respiratory to respiro-fermentative metabolism, which significantly lowered protein production in *H. polymorpha* RB11 conphys due to repression of the FMD promoter by extracellular ethanol. However, protein concentrations in the cultivation supernatants were sufficient for reproducible isotope mapping in amino acids from secreted protein at all applied dilution rates. Figure 8 displays the molar fractions of labeled (m + 1) and unlabeled (m + 0) m-57 amino acid fragments from continuous cultivations with 5 g L^−1^ 100% (mol/mol) 1-^13^C glucose at dilution rates of D = 0.05 h^−1^ (fully respiratory metabolism) and D = 0.2 h^−1^ (respiro-fermentative metabolism).

**Figure 9:**
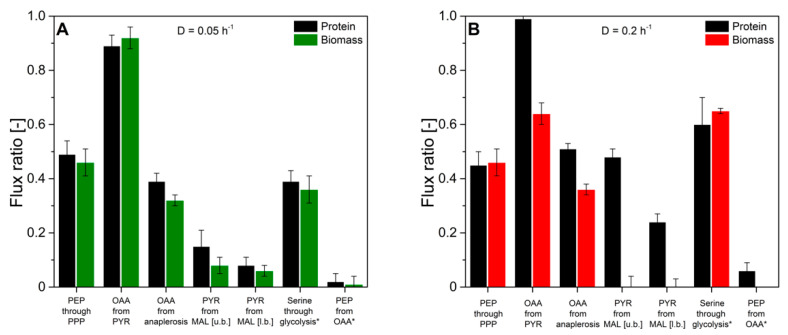
Comparison of intracellular flux ratios of *H. polymorpha* RB11 conphys cultivated at dilution rates of (**A**) D = 0.05 h^−1^ and (**B**) D = 0.2 h^−1^. Flux ratios were determined based on isotope mapping in amino acids from secreted protein and biomass with METAFoR. (PEP = phosphoenolpyruvate, PPP = pentose phosphate pathway, OAA = oxaloacetate, PYR = pyruvate, MAL = malate, u.b. = upper bound, l.b. =lower bound).

Next, we evaluated the flux probe concept based on the calculated intracellular flux ratios in *H. polymorpha* RB 11 at dilution rates of D = 0.05 h^−1^ and D = 0.2 h^−1^ (see Figure 9). As can be seen, the molar fractions of labeled and unlabeled amino acid m-57 fragments from secreted protein and biomass-bound protein were in excellent agreement within the range of the standard deviation at a dilution rate of D = 0.05 h^−1^. The maximal measured differences from three independent measurements were below 2%. At a dilution rate of D = 0.2 h^−1^, divergence in isotope abundance in the respective amino acid fragments increased to a maximal value of 5% for phenylalanine, aspartate, and glutamate and 7% for alanine, but was below 1% for glycine, valine, and serine. The extent of the detected deviations in amino acids labeling abundance between secreted protein and biomass-bound protein positively correlated with the applied dilution rate throughout this study. Virtually identical tendencies were observed for both experimental series using 100% (mol/mol) 1-^13^C glucose, as well as a mixture of 20% (mol/mol), U-^13^C glucose, and 80% (mol/mol) naturally labeled glucose.

The obtained intracellular flux ratios were highly similar for both secreted protein and biomass-bound protein at a dilution rate of D = 0.05 h^−1^, which agrees with the manually processed isotope abundance data. The only significant difference was observed for the upper bound of the malate to pyruvate flux ratio with a deviation of 48%. At a dilution rate of D = 0.2 h^−1^, the differences in amino acid isotope abundance from secreted protein and biomass-bound protein were directly reflected in the calculated intracellular flux ratio distributions with differences from 2% up to 55%. Thus, the flux probe concept could be validated for low dilution rates in continuous glucose-limited chemostats in metabolic and isotopic steady-state. Interestingly, the differences in isotope abundance could be quantitatively reproduced in several different experimental series. This points to a mechanistic effect responsible for the observed deviations in METAFoR analyses of flux probe protein and biomass-bound protein.

### 3.4. Dynamic ^13^C-Labeling Experiments in Metabolic Steady-State and Isotopic Nonstationary State

**Figure 10:**
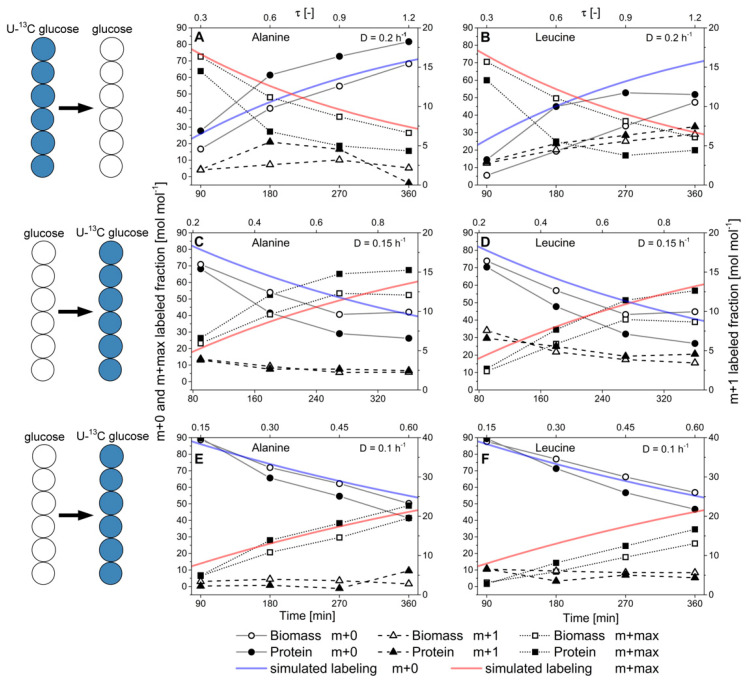
Labeling dynamics in *H. polymorpha* RB11 conphys in metabolic steady-state and isotopic nonstationary state at different dilution rates. Depicted are the molar fractions of the m + 0, m + 1, and m + max mass isotopomers into the m-57 fragment of pyruvate-borne alanine and leucine upon the change from uniformly-labeled ^13^C glucose to naturally labeled glucose or vice versa. Labeling dynamics of (**A**) alanine and (**B**) leucine under non-stationary isotopic conditions upon the change from 100% (mol/mol) U-^13^C glucose to 100% (mol/mol) naturally labeled glucose at a dilution rate of D = 0.2 h^−1^. Labeling dynamics of (**C**) alanine and (**D**) leucine under non-stationary isotopic conditions upon the change from 100% (mol/mol) naturally labeled glucose to 100% (mol/mol) U-^13^C glucose at a dilution rate of D = 0.15 h^−1^. Labeling dynamics of (**E**) alanine and (**F**) leucine under non-stationary isotopic conditions upon the change from 100% (mol/mol) U-^13^C glucose to 100% (mol/mol) naturally labeled glucose at a dilution rate of D = 0.1 h^−1^. M + 0 denotes the completely unlabeled carbon backbone, m + 1 the single-labeled amino acid backbone, and m+max the fully labeled backbone with ^13^C-isotopes. For alanine and leucine, m+max corresponds to the mass isotopomers m + 4 and m + 5, respectively. τ [-] represents the normalized residence time.

To elucidate the mechanistic principles behind the observed differences regarding isotope labeling in the flux probe protein, a kinetic analysis of isotope incorporation and drainage in amino acids from secreted protein and biomass-bound protein was performed. In order to do so, the carbon source was changed from 100% (mol/mol) naturally labeled glucose to 100% (mol/mol) U-^13^C glucose or vice versa in continuous glucose-limited cultivations at different dilution rates and the development of isotope accumulation or depletion in the carbon backbone of nine amino acids was followed with temporal resolution. Based on the obtained data, labeling kinetics (=the rate of isotope incorporation into biomass and secreted protein) in isotopic non-stationary, but metabolic steady state could be determined. These analyses granted access to intracellular amino acid and protein turnover dynamics and the usage of the intracellular amino acid pools for the synthesis of biomass-bound whole-cell protein and strongly overproduced and secreted heterologous phytase a flux probe protein. Isotope labeling kinetics of alanine and leucine in secreted protein and biomass-bound protein upon change of the carbon source labeling at different dilution rates are illustrated in Figure 10. In order to quantitatively assess labeling kinetics, the measured data were also compared to idealized, simulated isotope accumulation or depletion according to first-order kinetics. Ideal labeling kinetics can be described as a function of the specific growth rate µ respectively the dilution rate D as Nanchen et al. proposed [28].

(1) $$f_{label}\;\left(µ\right)=\;\zeta_{label}\;\times \left(1-e^{-µ\;\times \;t}\right)$$

with $f_{label}$ (mol mol^−1^) denoting the molar fraction of labeled amino acids after time t (h), $\zeta_{label}$ (mol mol^−1^) the molar fraction of isotope-labeled carbon source, and the specific growth rate µ (h^−1^).

Generally, faster turnover of ^13^C-isotopes was detectable in the amino acid backbones of the secreted flux probe protein than biomass-bound whole-cell protein. This behavior was consistently observed for all nine analyzed amino acids, regardless of the applied dilution rate or change of glucose labeling. The labeling kinetics of the m + 0 and m + max fragments of the analyzed amino acids originating from biomass-bound protein could be appropriately described by growth rate-dependent first-order kinetics. At the same time, the rate of isotope turnover in the flux probe was significantly higher than the simulated, growth-rate dependent values.

**Figure 11:**
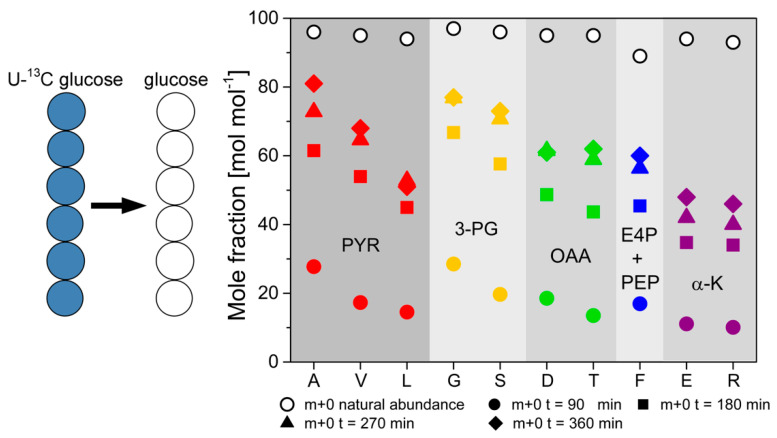
^13^C-labeling dynamics of 10 different amino acids in secreted phytase from *H. polymorpha* RB11 conphys cultivated in glucose-limited continuous cultivations. The dynamics were measured under non-stationary isotopic conditions upon the change from 100% (mol/mol) U-^13^C glucose to 100% (mol/mol) naturally labeled glucose at a dilution rate of D = 0.2 h^−1^. The amino acids are classified according to their precursor metabolites from the central carbon metabolism. (A = alanine, G = glycine, V = valine, S = serine, T = threonine, F = phenylalanine, D = aspartate, E = glutamate, L = leucine, R = arginine).

This effect was pronounced at a high dilution rate of D = 0.2 h^−1^, while the labeling differences decreased along with reduced dilution rate, and differences in isotope labeling between secreted protein and biomass-bound protein vanished. These results suggest generally faster processing and turnover of secreted protein from newly synthesized amino acids. In contrast, isotope turnover was retarded in biomass-bound protein due to its extended intracellular half-life, involving degradation and reassembly from amino acid pools. Notably, turnover kinetics of the different amino acids exhibited different tendencies towards isotope incorporation or drainage. They were primarily determined by the respective precursor metabolites rather than the abundance of the respective amino acids in the biomass-bound protein and secreted protein as the flux probe (see Figure 11).

In summary, the comparative kinetic analysis of isotope turnover in amino acids of secreted, recombinant protein and biomass-bound protein disclosed that information from ^13^C-isotope-labeled glucose is significantly faster incorporated into an overproduced and secreted heterologous protein than into biomass-bound protein.

## 4. Discussion

Here we present the application of a secreted protein as an alternative information carrier for ^13^C-based metabolic flux analysis apart from biomass-bound whole-cell protein. To evaluate the flux probe concept, we employed *H. polymorpha* RB11 conphys as one of the most efficient producers of extracellular protein in discontinuous and continuous cultivation set-ups. Furthermore, we developed a sample processing procedure that enabled consistent isotope mapping in the amino acids of the extracellular protein via GC-MS analysis. As expected, the flux probe was not applicable to the acquisition of isotope patterns in batch cultivation set-ups. The determined intracellular flux ratios significantly deviated between secreted protein and biomass-bound protein as an information source for isotope mapping. This observation could be attributed to the extracellular accumulation of averaged isotope labeling information, as secreted protein that did not reflect the usage of the actual metabolic network in *H. polymorpha* RB11 conphys [29,30]. Since biomass-bound protein is largely produced from de novo synthesized amino acids due to proliferation and intracellular protein turnover, the current metabolic pathway usage, reflected in ^13^C-isotope labeling patterns, is continuously updated in proteinogenic amino acids, which is not the case in secreted protein [31]. Provided that the accumulation of extracellular information in the form of the secreted protein was the mere source for the observed analytical bias, the flux probe concept was assumed to be applicable in continuous cultivation set-ups. In continuous chemostats, accumulation effects are typically minimized since secreted substances, like the extracellular phytase, are perpetually removed by the medium outflow. This, in turn, circumvents extensive accumulation of unspecific averaged isotope patterns in the cultivation supernatants. Indeed, at a high dilution rate of D = 0.2 h^−1^, amino acid isotope mapping in metabolic and isotopic steady-state in the flux probe protein yielded virtually identical intracellular flux ratio distributions as biomass-bound protein. Presumably, the high rate of protein exchange in the cultivation supernatant, accompanied by high specific growth rates, led to comparable isotope patterns in both secreted and biomass-bound protein. As a proof of concept, the application of a secreted protein as a flux probe for ^13^C-based studies on microbial metabolism in *H. polymorpha* RB11 conphys could be successfully validated. Although extracellular phytase titers were generally low due to reduced specific protein productivity due to glucose-repression of the employed FMD promoter system at the applied dilution rate. Protein titers were close to the limit of mass isotopomer quantification of the employed GC-MS analytics. However, at a reduced dilution rate of 0.05 h^−1^, unexpected differences in intracellular flux ratio distributions were determined with the flux probe compared to protein from biomass. Since the continuous cultures were in isotopic and metabolic steady state, the intracellular pathway usage was assumed to remain unchanged at a fixed dilution rate, making the observed differences difficult to interpret. Analytical issues can be excluded as a reason for that since isotope mapping of several independent biological and technical replicate yielded consistent results. This indicated that biological reasons were responsible for the observed deviations. Notably, the specific protein productivity increased eightfold compared to continuous cultures at D = 0.2 h^−1^, which was induced by relieved FMD promoter glucose-repression. As a result, more protein accumulated due to increased protein production, and fourfold slower protein removal from the cultivation supernatant was present due to the extended residence time. Moreover, along with the reduced dilution rate, the specific growth rate was also reduced fourfold, which was in turn leading to slower intracellular protein turnover in the present biomass as a possible reason for the determined discrepancies [32]. Thus, physiological changes resulting in altered pathway usage patterns should be immediately reflected in the ^13^C-labeling patterns of the secreted flux probe protein. At the same time, the incorporation of labeling into the biomass is delayed due to the intracellular protein half-life (mean half-life of 43 min in budding baker’s yeast) [33]. Such intracellular effects might become dominant in the low dilution rate scenario, with high production rates of secreted protein and delayed biomass protein turnover and obscure physiological changes that take place on relatively short time scales, e.g., synchronized glycolytic respiratory oscillations in yeast and cell cycle effects [34,35,36]. It has been also demonstrated that intracellular free amino acids pools are affected by such oscillations [37]. Compartmentalization and the resulting sub-cellar proteome regimes in yeast, like the mitochondrial, nucleus, or intermembrane proteome, might also play an important role in delaying effects in terms of the incorporation kinetics of ^13^C-labeling information into intracellular protein. Our comparative kinetic analysis of isotope incorporation and depletion in secreted protein and biomass-bound protein further substantiates this suggestion. These analyses indicated that overproduced and secreted phytase protein is assembled from de novo synthesized amino acids, bypassing retarding detours through the intracellular protein production and destruction machinery in *H. polymorpha* RB11 conphys [38,39]. This aspect might be vital for studies that rely on nonstationary ^13^C-labeling experiments at short time scales to resolve intracellular metabolic dynamics [40,41]. Different labeling kinetics could also be observed among the respective amino acids in the secreted protein. Here, the respective precursor metabolites of the respective amino acid families were identified as a major determinant for the kinetics of ^13^C-labeling incorporation and depletion in the amino acids of the secreted protein.

### The Single-Cell Flux Probe: Opportunities and Challenges

The presented study also enables us to conclude the future applicability of the flux probe concept for single-cell analyses. In a continuous single-cell cultivation system, like the microfluidic Envirostat, a flux probe protein is principally capable of delivering accurate ^13^C-labeling information that reflects the actual usage of the intracellular metabolic network. Accumulation effects observed in population-based batch cultivations and continuous cultivations at low dilution rates are a priori excluded since the cell is continuously perfused with fresh medium and secreted protein is immediately removed. Moreover, physiological changes due to environmental changes are minimized to the greatest possible extent. Therefore, it is instead a question of analytical sensitivity to determine the isotope patterns in the amino acids from secreted single-cell flux probe protein via mass spectrometry. The current generation of high-end mass spectrometry devices combined with microfluidics can distinguish high mass compounds like peptides at femtomolar concentration levels [42]. However, such studies are still in the proof-of-concept stage and mainly deal with mammalian cells that exhibit significantly higher target analyte concentrations than microbial cells.

Nevertheless, we took the first steps towards evaluating the single-cell flux probe concept in single yeast. We harnessed with MALDI-MS the same analytical principle as Urban et al. to successfully detect ^13^C-labeled ATP in single yeast cells to determine natural isotope abundance in five different amino acids [19,43]. Reliable quantification of the natural mass isotopomers in, e.g., phenylalanine was possible due to a concentration of 100 nM in an aqueous solution. This constitutes an excellent LOD for mass spectrometric analysis of small molecules like amino acids but still falls short by at least two orders of magnitude (expected in single-cell cultivation supernatant C_phenylalanine_ ≈ 5 nM) to the calculated averaged amino acid productivities from the secreted protein of an individual, trapped recombinant *H. polymorpha* cell. In addition, one should also consider that *H. polymorpha* RB11 conphys is one of the most efficient protein production platforms among the many known and engineered microorganisms. These first attempts in the field of single-cell fluxomics demonstrate the current analytical sensitivity of mass spectrometry as the primary limit for ^13^C-based flux probe studies at a single-cell level.

## 5. Conclusions

The presented study demonstrates the first application of a secreted protein probe as a non-disruptive ^13^C-isotope carrier for flux ratio analysis. We could successfully demonstrate the flux probe concept to be valid and yield similar intracellular flux distributions as with biomass under certain cultivation conditions; however, new and fundamental questions concerning intracellular processing of ^13^C-isotopes arise from our results. Delayed isotope incorporation into biomass is typically disregarded to justify low starting cell concentrations in batch cultivations or extended steady-states in chemostats, which should ultimately ensure sufficient dilution of outdated labeling information in biomass-bound protein. However, the differences in ^13^C-labeling information in secreted flux probe protein and biomass observed during this study suggest reassessing these aspects. The flux probe concept might become especially useful as a complementary analytical approach for dynamic ^13^C-labeling experiments in nonstationary isotopic states combined with ultra-short sampling intervals. In such set-ups, the increased rates of isotope incorporation into overproduced and secreted protein would be beneficial for resolving intracellular fluxome dynamics at high temporal resolution. If future progress in the analytical sensitivity of mass spectrometry will be made, the flux probe concept would also pose a promising approach for ^13^C-based flux ratio analysis with single-cells in combination with robust microfluidic systems like the Envirostat.

In summary, we introduced a new analytical concept that grants new insight into intracellular dynamics and enables ^13^C-based flux analysis without the need to consume any biomass.

## Supplementary Materials

The following are available online at https://www.mdpi.com/article/10.3390/ijms22179438/s1.
