# Supplementary material for: The Metabolic Flux Probe (MFP)—Secreted Protein as a Non-Disruptive Information Carrier for 13C-Based Metabolic Flux Analysis
Source: Int J Mol Sci. 2021 Aug 30;22(17):9438. doi: 10.3390/ijms22179438 (PMC8430588; doi:10.3390/ijms22179438)
Supplement: Supplementary file 1 [file ijms-22-09438-s001.zip › ijms-1321705-supplementary.pdf]

Supplementary information

The metabolic flux probe (MFP) - secreted protein as a non-disruptive information carrier for ^{13}C-based metabolic flux analysis

Supplementary Table S1: List of abbreviations.

ATP	Adenosine triphosphate
BSA	Bovine serum albumine
CDW	Cell dry weight
CoA	Coenzym A
cyt.	cytosolic
FAD	Flavin adenine dinucleotide
FMD	Format dehydrogenase
GAP	Glyeraldehyde 3-phosphate
GC	Gas chromatography
HPLC	High pressure liquid chromatography
kDA	kilo Dalton
LOC	Lab-on-a-chip
MBDSTFA	N-methyl-N-tert-butyldimethylsilyl-trifluoroacetamide
MES	2-(*N*-morpholino)ethanesulfonic acid
METAFoR	Metabolic flux ratio
MFA	Metabolic flux analysis
MOX	Methanol oxidase
MS	Mass spectrometry
MW	Molecular weight
MWCO	Molecular weight cut off
myt.	mytochondrial
NADH	Nicotinamide adenine dinucleotide
NADPH	Nicotinamide adenine dinucleotide phosphate
OD	Optical density
OD_{600}	Optical density at a wavelength of 450 nm
pO_2	Partial pressure of O2
rpm	Rounds per minute
SDS-PAGE	Sodium dodecyl sulfate polyacrylamide gel
SIM	Selective ion monitoring
TCA	Tricarboxylic acid cycle
TEMED	N,N,N’,N’-Tetramethylethan-1,2-diamin
YPD	Yeast extract peptone dextrose

Supplementary Table S2: Metabolic network of *H. polymorpha*.

R1	Glucose + ATP	→	Glucose 6-phosphate
R2	Glucose 6-phosphate	→	Pyridoxale 5-phosphate + 2*NADPH +CO_2
R3	Glucose 6-phosphate	↔	Fructose 6-phosphate
R4	Fructose 6-phosphate + ATP	→	2*Triose 3-phosphate
R5	2*Pyridoxale 5-phosphate	↔	Sedoheptulose 7-phosphate + Triose 3-phosphate
R6	Pyridoxale 5-phosphate + Erythrose 4-phosphate	↔	Fructose 6-phosphate + Triose-3-phosphat
R7	Sedoheptulose 7-phosphate + Triose 3-phosphate	↔	Erythrose-4-phosphate + Fructose 6-phosphate
R8	Triose 3-phosphate	→	Serine + NADH
R9	Serine + NADH	→	Glycine + C1
R10	Glycine + C1	→	Serine
R11	C1 + CO_2 + NADH	→	Glycine
R12	Triose 3-phosphate	↔	Phosphoenolpyruvate + ATP + NADH
R13	Phosphoenolpyruvate	→	cyt. Pyruvate + ATP
R14	mit. Pyruvate	→	mit. AcCoA + CO_2 + NADH
R15	mit. Oxalacetate + mit. AcCoA	→	Citrate
R16	Citrate	→	Oxoglutarate + CO_2 + NADH
R17	Oxoglutarate	→	Succinate + CO_2 + 0.5*ATP + NADH
R18	Succinate	↔	Fumarate + NADH
R19	Malate	↔	mit. Oxalacetat + NADH
R20	Fumarate	↔	Malate
R21	Malate	↔	mit. Pyruvat + CO_2 + NADPH
R22	cyt. Oxalacetate + ATP	→	Phosphoenolpyruvate + CO_2
R23	cyt. Pyruvate + CO_2 + ATP	→	cyt. Oxalacetate
R24	Acetate + 2*ATP	→	cyt. AcCoA
R25	Acetaldehyde	↔	Acetate + NADPH
R26	Acetaldehyde + NADH	→	Ethanol
R27	Triose-3-phosphate + NADH	→	Glycerol
R28	cyt. Oxalacetate	→	mit. Oxalacetate
R29	mit. Oxalacetate	→	cyt. Oxalacetate
R30	cyt. AcCoA	→	mit. AcCoA
R31	cyt. Pyruvate	→	mit. Pyruvate
R32	cyt. Pyruvate	→	Acetaldehyde + CO_2
R33	NADPH	→	NADH
R34	O_2 + 2*NADH	→	2*P/O*ATP

Supplementary Table S3: Intracellular flux ratios in *H. polymorpha* implemented in the NETTO module of FiatFlux.

cytosolic oxalacetate from cytosolic pyruvate:

$$\frac{R23}{R23 + R29}$$

mitochondrial oxalacetate from anaplerosis:

$$\frac{R28}{R28 + R19}$$

phosphoenolpyruvate from cytosolic oxalacetate

$$\frac{R22}{R22 + R12}$$

serine from glycolysis

$$\frac{2 \cdot R4 - 2 \cdot R6 - 2 \cdot R7}{2 \cdot R4 + R7 + R6}$$

mitochondrial pyruvate from malate (upper bound)

$$\frac{R21}{R21 + R31}$$

mitochondrial pyruvate from malate (lower bound)

$$\frac{R21}{R21 + R31}$$

serine from glycine

$$\frac{R10}{R10 + R8}$$

phosphoenolpyruvate through pentose-phosphate pathway

$$\frac{R5 + 3 \cdot R6 + 2 \cdot R7}{2 \cdot R4 + R5 + R6}$$
